# Impact Assessment of Pharmaceutical Care in the Management of Hypertension and Coronary Risk Factors after Discharge

**DOI:** 10.1371/journal.pone.0155204

**Published:** 2016-06-15

**Authors:** Maurílio de Souza Cazarim, Osvaldo de Freitas, Thais Rodrigues Penaforte, Angela Achcar, Leonardo Régis Leira Pereira

**Affiliations:** 1 Department of Pharmaceutical Sciences, School of Pharmaceutical Sciences of Ribeirão Preto, University of São Paulo, Ribeirão Preto, state of São Paulo, Brazil; 2 Department Medicine, Pharmacy School, Federal University of Bahia, Salvador, state of Bahia, Brazil; 3 Department of Social Medicine, School of Medicine of Ribeirão Preto, University of São Paulo, Ribeirão Preto, state of São Paulo, Brazil; Hospital de Clínicas de Porto Alegre, BRAZIL

## Abstract

**Introduction:**

Almost 50% of the 17.5 million deaths worldwide from cardiovascular disease have been associated with systemic arterial hypertension (SAH). Into this scenario, Pharmaceutical Care (PC) has been inserted in order to improve the management of SAH and reduce its risks.

**Objective:**

To evaluate the outcomes and healthcare assistance achieved after discharge of hypertension patients from the PC program.

**Methods:**

This is a quasi-experimental study with historical controls. Retrospective data collection from 2006 to 2012 was begun in 2013 and included a PC program performed over one year. PC was performed in two basic units of the public health system in Ribeirão Preto-SP, Brazil, where the pharmacist followed up 104 hypertensive patients. The clinical indicators of systolic (SBP) and diastolic blood pressure (DBP), triglycerides, total-cholesterol, high and low density lipoprotein cholesterol were collected, as well as care indicators related to the number of consultations (basic, specialized and emergency care) and antihypertensive drugs used. The coronary risk of patients by the Framingham risk score was also calculated. For the analysis, the data were divided into three periods, 2006–2008 as pre-PC, 2009 as PC and 2010–2012 as post-PC.

**Results:**

In the pre-PC period, 54.4%, 79.0% and 27.3% of patients presented satisfactory levels of SBP, DBP and total-cholesterol, respectively. In the post-PC period, the percentages were 93.0% for SBP and DBP [p <0.001] and 60.6% for total-cholesterol [p <0.001]. The average number of consultations per patient/year in primary care was 1.66 ± 1.43 and 2.36 ± 1.73, [p = 0.012]; and for emergency care was 1.70 ± 1.43 and 1.06 ± 0.81, [p = 0.002] in the pre-PC and post-PC periods, respectively. The pre-PC Framingham risk in the last year was 14.3% ± 10.6 and the average post-PC was 10.9% ± 7.9.

**Conclusion:**

PC was effective in the control of blood pressure and total-cholesterolafter discharge of the hypertensive patients from PC program.

## Introduction

Systemic arterial hypertension (SAH) is a morbidity considered a major risk factor for cardiovascular disease (CVD) [1]. In 2012, almost 50% of the 17.5 million deaths worldwide from CVD were associated with SAH [2, 3]. In Brazil, SAH affects about one third of the population, reaching over 50% in old age [4]. The greatest risk factor for myocardial infarction, heart failure and stroke is poor blood pressure control [5]. The increment of only 10 mmHg in systolic blood pressure (SBP) can increase by up to 25% the risk of developing CVD. In addition, only 30% of hypertensive patients have blood pressure at appropriate levels [6], which shows that health systems together with health technologies used for the treatment of hypertensive patients are not effective to maintain recommended blood pressure levels.

The consequence of this has been the impact on access to health and the quality of the public health system (PHS) in Brazil. The current public health policy in Brazil has been defined as a duty of the State and a right of the citizens. In this sense, the PHS was structured to provide care for the entire Brazilian population, organizing for equity and comprehensive care, and ensuring the participation of society. This complex model of health requires planning and results for its efficiency. The results of the control of hypertension and its complications have damaged the flawless operation of this system due to required planning and resources, and have not generated improvement of outcomes [7, 8].

An alternative considered as a health technology to provide better prospects in the control of chronic diseases is pharmaceutical care (PC) [9, 10, 11, 12, 13]. PC is the professional practice that requires clinical and humanistic training of pharmacists in search of personalized support to patients. This practice involves activities such as consultations, interventions, and monitoring records, following a therapeutic plan with clear goals to discharge the patient [14]. PC has proven effective in the management of hypertension and in reducing the risk of CVD [11, 15, 16, 17, 18].

These achievements evidence the pharmacist as a professional for insertion into health teams in Brazil [19, 20]. This setting in health care allowed the pharmacist to be inserted into the Brazilian Hypertension Guidelines in collaboration with multidisciplinary care of hypertensive patients [4, 21]. However, public health policies do not have a guideline or a consolidated model for the development of PC in the PHS. An important issue to encourage the implementation of PC in the PHS is the visualization of results obtained from PC after discharge of patients. In this context, this study aimed to evaluate the outcomes and healthcare assistance achieved by a PC program over three years after discharge of the hypertensive patients.

## Subjects and Methods

This is quasi-experimental study with historical controls, “S1 Appendix”. Intervention was performed by the PC program for a period of one year. This PC program was conducted by one pharmacist in two units of primary health of the PHS in Ribeirão Preto-SP, Brazil. Individuals diagnosed with SAH and in medical care for the disease, aged over 20 years, users of the health unit within the PC program, and those who used at least one antihypertensive medication were included in the program. The program excluded patients who could not continue the planning of pharmaceutical consultations, pregnant women and those who had some kind of diagnosed cognitive impairment.

### Ethics

The Research Ethics Committee of the School of Pharmaceutical Sciences of Ribeirão Preto, University of São Paulo has approved this study, protocol No. 015/2013 and release No. 292 CEP/FCFRP; ruling No. 263665, CAAE 07709612.5.0000.5403 (*http*:*//plataformabrasil*.*saude*.*gov*.*br/login*.*jsf*).

The PC program is part of another study developed by a pharmacist [22] and had the approval of the Research Ethics Committee of the Health Center of the Ribeirão Preto Medical School, University of São Paulo, document 664/07/COORD. CEP/CSE-FMRP-USP-12/12/2007, protocol No. 256/CEP-CSE-FMRP-USP and release 054/2007. From August to November 2008 the patient was approached at the time of receipt of the drug in the health unit, and invited to participate in the PC program. If the patient met the inclusion criteria and did not meet any item in the exclusion criteria, they were invited to sign the Free and Informed Consent Terms, with guidance from the pharmacist of the program. From this moment the patient was included in the PC program. During 2009, from January to December, one pharmacist followed up 104 patients in this PC program. In 2013, data collection for this study was performed in order to gather the clinical and health care data of individuals monitored by PC. Thus, from January 2006 to December 2012 the data were collected through the patient record and computerized system.

### Pharmaceutical Care Program

The pharmaceutical consultations were based on the model of the Pharmacist’s Workup of Drug Therapy (PWDT) pharmacotherapeutic monitoring model [23]. Therefore, during 2009 each patient was scheduled to consult the pharmacist once a month during a year in the health units of the study. The first appointment was designated as a welcome consultation to obtain the medical history of the patient, demographic data, socioeconomic profile, lifestyle, eating habits and measures for cardiovascular risk profile.

Subsequent consultations followed the relevant activities of pharmacotherapeutic monitoring, considering blood pressure measurements and measures of cardiovascular risk, analysis of medicines and test results, health education with guidance on patient behavior regarding life habits, adherence to treatment, and if necessary interventions in pharmacotherapy. By analyzing the drugs used by the patient, the pharmacist verified the presence of possible drug related problems (DRP). On detecting a DRP, the pharmacist completed a form containing the breakdown of the drug in question (name, presentation, daily dose, dosage form and administration time), classified the DRP according to the PWDT, described the intervention to be performed and the proposed outcomes to be achieved with the intervention, established the form of monitoring of these results, designated the deadline for re-evaluation of the clinical outcome and filled in the field concerning the adherence to the intervention by the prescriber. In addition, the patient was informed and educated about the new behaviors in their pharmacotherapy when there was acceptance by the physician.

Biochemical parameters were analyzed using routine techniques adopted according to the specialized literature and established by usage at the Clinical Analysis Laboratory. Measurements of body weight (kg) and height (m) were obtained with a Filizola® brand anthropometric scale. Body Mass Index (BMI) was obtained by the ratio between weight and the square of height. Waist circumference was considered as half the distance between the last rib and the iliac crest between inhalation and expiration and was measured using a tape measure, according to the VI Brazilian Guidelines on Hypertension criteria. Blood pressure was monitored using a mercury column sphygmomanometer, and the measurements were standard and always performed in the same way and two measurements per patient were taken at each consultation [4].

The identification of problems related to drug therapy occurred during the monthly consultation, and the proposed classification was reasoned according to the PWDT model: needs additional pharmacological treatment; unnecessary pharmacological treatment; inadequate medicine; drug dose lower than needed; drug dosage higher than needed; adverse reaction to drugs; and inappropriate adherence to drug therapy [23].

Throughout the PC period the pharmacist worked on health education through informative lectures, educational materials on health, and guidance during the consultations. Adherence to drug therapy was also worked on. Both behaviors aimed to empower the patient about their treatment, educating them about their illnesses, severity, complications and care needed. These strategies had their results measured by the SF-36 instrument (The medical outcomes study 36-item short-form health survey), and the Morisky-Green instrument. The Morisky Medication Adherence Scale with four questions was used (MMAS-4). The SF-36 assesses quality of life of the patients and Morisky-Green assesses adherence to pharmacotherapy for patient self-reports on the use of medicines. Both instruments were applied twice for all patients, once during the first consultation and the other during the last consultation [24, 25].

### Indicators to assess the impact of Pharmaceutical Care

The indicators were defined according to the clinical and care data, considering primary outcome, blood pressure, secondary outcomes, plasma lipid levels, coronary risk, and care. The clinical indicators of SBP and DBP, triglycerides (TG), total cholesterol (TC) and fractions, low density lipoprotein (LDL), and high density lipoprotein (HDL) were considered. The care indicators used refer to the consultations and antihypertensive drugs. The consultations were classified into primary care (consultations with general practitioners and medical family health), emergency care (consultations by hypertensive crisis in emergency care) and specialized care (consultations with the cardiologist). Data on antihypertensive drugs provided by the PHS, part of the municipal list of standard drugs, were collected. Another indicator was coronary risk over ten years, which was calculated by the Framingham risk scale using the following variables: age, sex, blood pressure values, TC with HDL fractions, and comorbidities such as SAH, smoking and diabetes [26, 27, 28].

### Sample planning and sample of Pharmaceutical Care study

Prior to PC follow up, the sample planning was carried out, being based on statistical calculation for experimental studies, using the estimated population mean for variable blood pressure. The calculation was performed as n = [(Z_α/2_ x dp_Δ_) / Ɛ]^2^where: *Z*_*α/2*_ is the standard normal percentile for a significance level of 5%, i.e., a sensitivity of 0.05; *dp*_*Δ*_ is the sum of the standard deviations before and after the intervention; *Ɛ* is the accuracy, i.e. a potential clinically significant difference between before and after the intervention. From the literature the sum of the standard deviations equal to 30 mmHg and a clinically satisfactory reduction in SBP between 5–10 mmHg was considered. Thus, for an Ɛ value between 5–10 mmHg *n* was calculated as between 33 and 138 [11, 17, 29].

**Fig 1:**
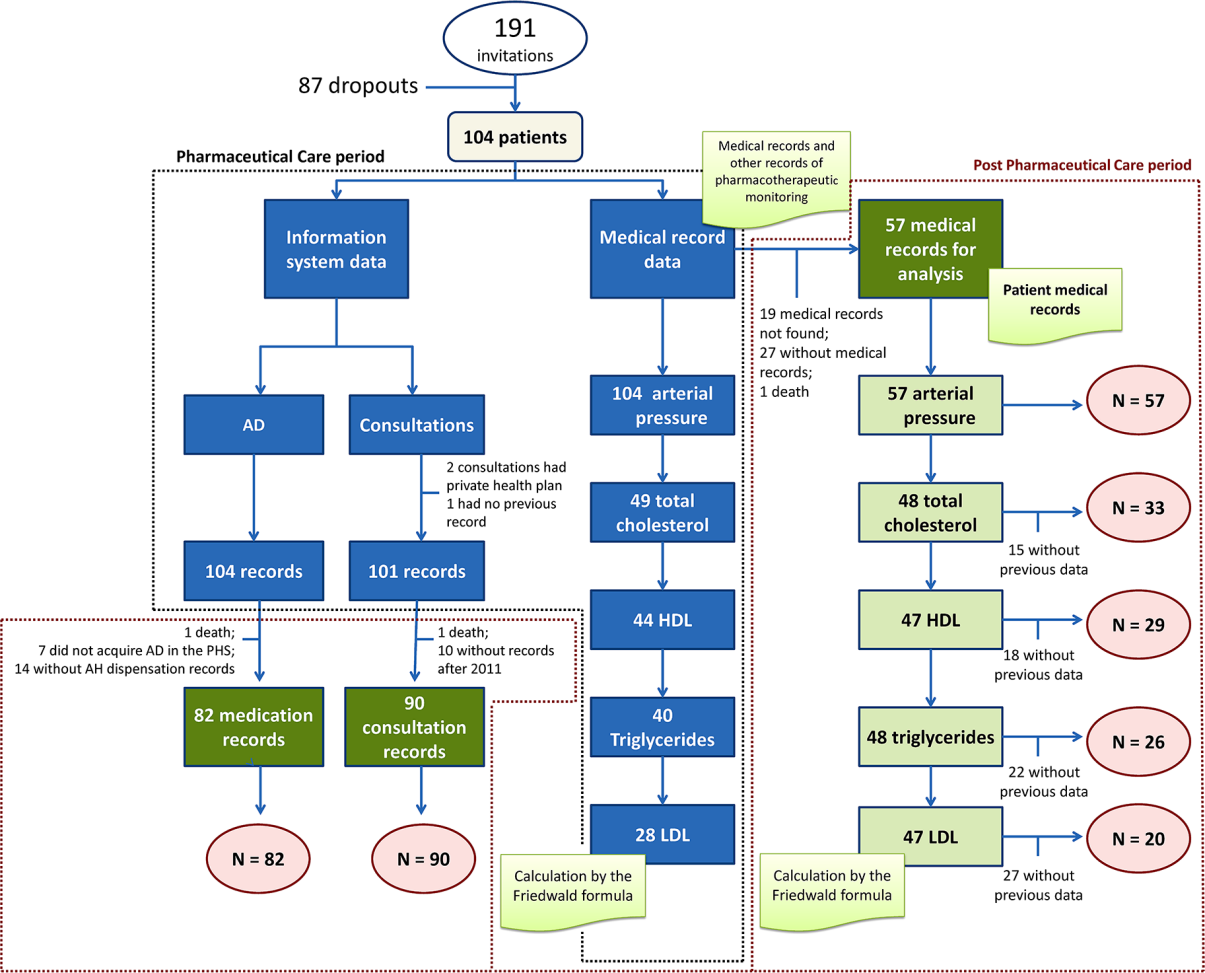
Flowchart of the stratified sample size for each variable analyzed. AD = Antihypertensive Drug; PHS = Public Health System; LDL = Low Density Lipoprotein; HDL = High Density Lipoprotein.

The method used to select the sample was convenience sampling, whereby 228 patients were invited and 191 patients were considered eligible in accordance with the inclusion criteria and 37 individuals were excluded because they did not fulfill the eligibility criteria for this study. The study of pharmaceutical follow up had no control group, being designed as before and after study. Thus, a stratification was performed on the sample size for each variable in order to analyze the data (Fig 1).

It is noteworthy that LDL was recorded in the medical records through calculation by the *Friedewald* formula, LDL = (TC—HDL)—(TG / 5) and where possible has been calculated in this way for records in which this information was missing.

### Statistics

For the analysis of clinical indicators, the data were categorized as satisfactory and unsatisfactory. For this we used the reference values for blood pressure according to the North American guideline regarding the VIII Joint National Committee 2014 [30]. The guideline sets the satisfactory values as blood pressure <150x90 mmHg for hypertensive individuals over 60 years of age without diabetes and without chronic kidney disease, and blood pressure <140/90 mmHg for individuals over 18 years of age with hypertension, with or without diabetes and chronic kidney disease. The reference levels for cholesterol were based on the dyslipidemia guideline of the American Association of Clinical Endocrinologists 2012 [31]. According to the clinical stage of coronary risk estimated by the Framingham risk score, patients were classified into high, intermediate and low risk. Additionally, for each risk stage a reference value was assigned as satisfactory, as established by the guideline. For TC, high risk is considered as <200 mg/dL, and between 200–239 mg/dL for intermediate and low risk; for LDL, <100 mg/dL, <130 mg/dL, and <160 mg/dl respectively for the three risk stages; for HDL >40 mg/dL for men and >50 mg/dL for women; for TG <150 mg/dL for high risk, and between 150–199 mg/dL for the intermediate and low and risk stages [31].

In relation to the analysis of the care indicators, it is emphasized that the drugs were classified according to the designation ATC/DDD of the World Health Organization. This classification was useful to obtain the maximum doses (max dose) for the pharmacotherapeutic power of each drug for the treatment of SAH. It was then possible to find the least, or greatest common divisor, of the maximum doses of all antihypertensive drugs and divide by the maximum dose of each drug, finding a fixed amount for each drug. This fixed value found for each drug was multiplied by the consumption of the respective medication for each patient. This adjustment model made possible the comparison among different drugs of the study (Table 1).

**Table 1:** Example of adjustment model of data elaborated in this study to analyze the antihypertensive drugs, therapeutic doses for comparison (TDC model).

3600 = LCM found considering all antihypertensive drugs		
Medication:	Hydrochlorothiazide	Amlodipine
Dose max:	50 mg	10 mg
Fixed amount:	3600 / 50 = 72	3600 / 10 = 360
Patient consumption:	25 mg per day	5 mg per day
		
Adjustment:	25mg x 72 = **1800mg**	5mg x 360 = **1800mg**

**Note Table 1:** This dose adjustment method for comparison and analysis of drug utilization is in the process of intellectual property registration. This process also includes the adjustment by greatest common divisor. Thus, this method cannot be used for commercial purposes. In addition, it is authorized for academic publishing when the authors are cited, “therapeutic doses for comparison model (TDC model) by Cazarim & Pereira”, in this paper, (by USP Innovation Agency).

The inferential statistical analysis was performed using *Statistical Analysis System* (SAS) version 9.2 and to develop the graphs of the statistical analysis the *GraphPad Prisma* version 5 was used. For hypothesis testing a 5% significance level was considered. Importantly, the data were divided into three periods for analysis: from 2006 to 2008, defined as pre-PC; in 2009 defined as PC; from 2010 to 2012 defined as post-PC.

The inferential statistic was based on paired data, this means relating to the data of the same individuals for analysis at different time points, because of this there were no potential confounders. Thus, for the clinical indicators the *Cochran Q test* was performed to compare categorical variables. This analysis tested the hypothesis that modification of outcomes is associated with PC. For this analysis the chi-square distribution for 2 degrees of freedom was considered, which presents the threshold for analysis of the results of 5.99. As for the care indicators, consumption of antihypertensive drugs in mg, a variable of continuous quantitative type, was analyzed by *one-way ANOVA* for repeated measures with the *Bonferroni post-test* to compare groups of data. The purpose was to test the hypothesis that there is a change in average antihypertensive drugs consumption caused by this model of PC. Regarding the number of consultations, corresponding to the variable of discrete quantitative type, the analysis of Friedman's variance with *Dunn’s post-test* was performed to compare groups of data. This analysis aimed to test the hypothesis that the number of consultations is modified by the PC model of this study. In the Framingham risk analysis, *one-way ANOVA* for repeated measures with the *Bonferroni post-test* was also conducted to verify the change in coronary risk among the years studied and test the hypothesis that the PC developed in this study is able to interfere in coronary risk over a period of ten years.

## Results

The results of this study derive from a sample, making inference to a population of hypertensive patients with cardiovascular risk and sociodemographic profile presented in Table 2 [4, 32].

**Table 2:** Profile of hypertensive patients at the start of the Pharmaceutical Care.

Sociodemographic Profile				Cardiovascular Risk Profile			
		n (104)	%			n (104)	%
**Sex**				Obesity*(BMI ≥30 Kg/m^2^)	Yes	56	53,9
Male		26	25,0		No	48	46,2
Female		78	75,0				
**Age**							
30–40 years		2	1,9	**Dyslipidemia**	Yes	28	26,9
41–59 years		35	33,7		No	76	73,1
>60 years		67	64,4				
**Skin Color**							
White		58	55,8	**Family History**	Yes	14	13,5
Black		8	7,7		No	90	86,5
Mixed		38	36,5				
**Education**							
Iliterate		8	7,7	**Smoking**	Yes	5	4,8
Primary Education		75	72,1		No	99	95,2
High School		17	16,3				
University		4	3,9				
**Ocupational Activity**		71	68,3	**Diabetes**	Yes	19	18,3
**Family Income**					No	85	81,7
up to 1 MW		7	6,7				
>1–2 MW		26	25,0				
>2–3 MW		29	27,9	Waist Circumference*	Changed	84	80,8
>3–5 MW		32	30,8		Not Changed	20	19,2
>5 MW		10	9,6				

* Source: VI Brazilian Guidelines on Hypertension (2010); BMI = Body Mass Index; Changed waist circumference values greater than 102 cm for men and 88 cm for women were considered; MW = Minimum Wage, household income was classified according to Brazilian Institute of Geography and Statistics—IBGE.

**Fig 2:**
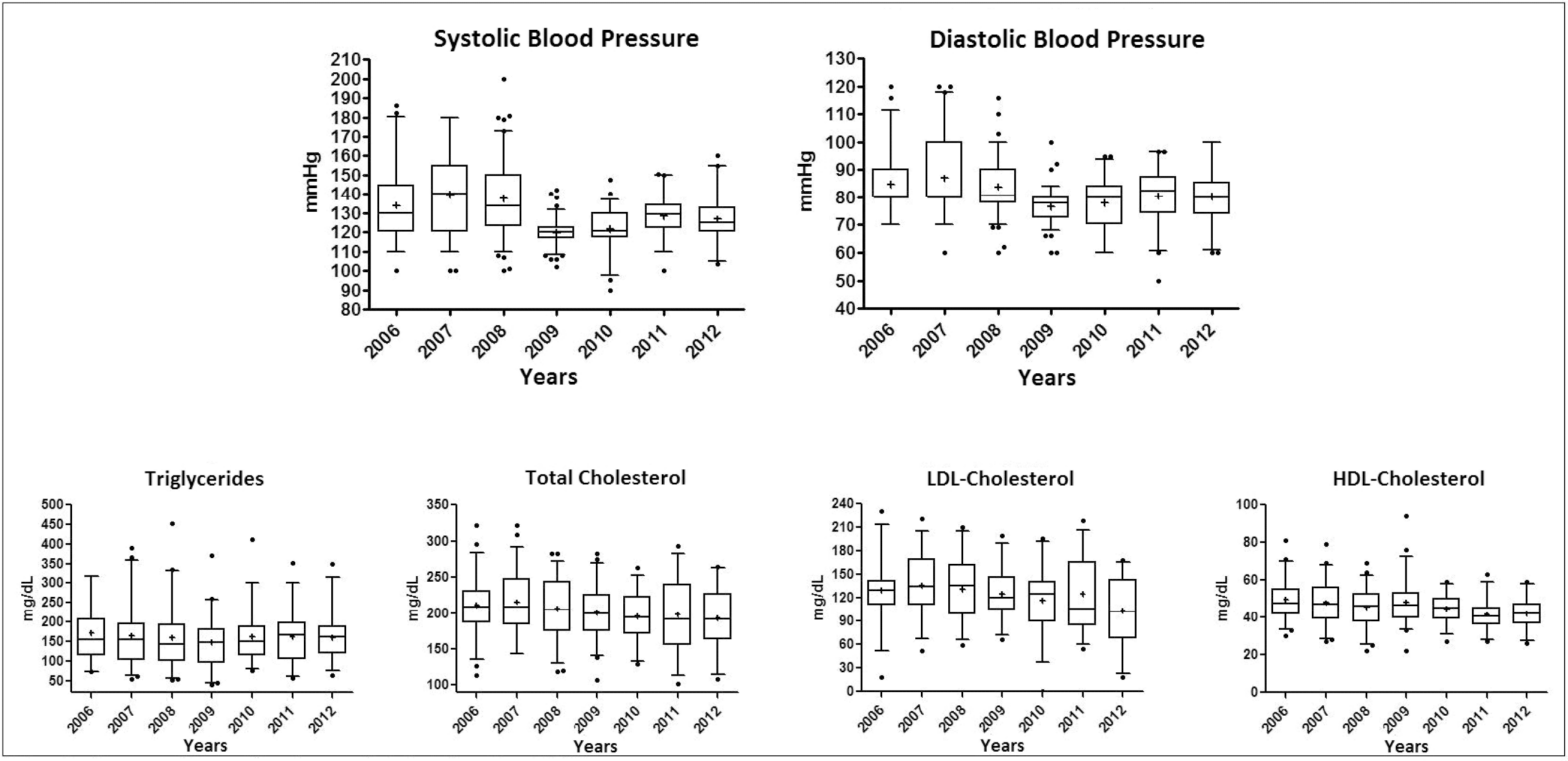
Distribution of clinical variables over seven years of follow-up of hypertensive patients considering the 5–95 percentile range. Considering the 5–95 percentile range for distribution; LDL = Low Density Lipoprotein; HDL = High Density Lipoprotein

Mean SBP and DBP in the three years of the pre-PC period were 134.0 mmHg ± 20.3 and 139.6 mmHg ± 20.5; 137.6 mmHg ± 19.5 and 84.7 mmHg ± 11.4; 87.0 mmHg ± 13.0 and 84.0 mmHg ± 10.1, respectively for 2006, 2007 and 2008. In 2009, with PC the average was 119.7 mmHg ± 7.3 and 76.7 mmHg ± 5.8 for SBP and DBP, respectively. For post-PC there were means equal to 121.5 mmHg ± 10.8; 128.2 mmHg ± 11.3; 126.8 mmHg ± 14.0 and 77.9 mmHg ± 8.7; 80.4 mmHg ± 9.8; 80.3 mmHg ± 9.7 for SBP and DBP in the three years, respectively. For TC the means 211.1 mg/dL ± 40.3; 215.6 mg/dL ± 42.7; 205.8 mg/dL ± 41.7 for 2006, 2007, 2008, respectively were observed. In 2009 TC was 200.2 mg/dL ± 37.8, and in the following years it was 195.8 mg/dL ± 35.8; 197.6 mg/dL ± 51.5; and 193.0 mg/dL ± 39.9, respectively. It can be noted that the distribution of the data on clinical variables was closer to the mean and median in 2009, representing greater homogeneity between the measures in this period (Fig 2).

Of 57 patients who had the SBP and DBP measures analyzed, 31 and 45 show satisfactory measures respectively. Between PC and post-PC, the number was 56 and 53 patients, with the Q statistic equal to 43.35 and 85.85, respectively. It can be noted as for TC, of the 33 patients analyzed with this indicator, the number of patients with a satisfactory value was 9 for pre-PC, and 17 and 20 in the PC and post-PC periods respectively. The value of the statistic Q equal to 14.92 was presented (Table 3).

**Table 3:** Proportion of patients with satisfactory outcomes for the three periods of analysis of the results.

Clinical Indicators		Pre-PC	PC	Post-PC	p value
	*n*	Satisfactory (%)	Satisfactory (%)	Satisfactory (%)	
**SBP**	*57*	31 (54,4%)	56 (98,2%)*	53 (93,0%)*	<0,001
**DBP**	*57*	45 (79,0%)	56 (98,2%)*	53 (93,0%)*	<0,001
**TG**	*26*	14 (53,8%)	11 (42,3%)	15 (57,7%)	0,236
**TC**	*33*	9 (27,3%)	17 (51,5%)*	20 (60,6%)*	<0,001
**LDL**	*20*	8 (40,0%)	11 (55,0%)	11 (55,0%)	0,325
**HDL**	*29*	6 (20,7%)	8 (27,6%)	5 (17,2%)	0,882

* Q Statistics for the Chi-Square > 5.99 (threshold to reject the null hypothesis); SBP = systolic blood pressure; DBP = diastolic blood pressure; TG = triglycerides; TC = total cholesterol; LDL = Low Density Lipoprotein; HDL = High Density Lipoprotein.

The average number of consultations in primary care was 1.66 ± 1.43 in the pre-PC period and 2.36 ± 1.73 in the post-PC. For the number of consultations in emergency care the average in the pre-PC period was 1.70 ± 1.38, and the PC and post-PC periods were 1.17 ± 1.29 and 1.06 ± 0.81, respectively (Table 4).

**Table 4:** Summary of measures and dispersion on consultation and consumption of antihypertensive drugs for the three periods of analysis of the results.

Care Indicators		Pre-PC		PC		Post-PC		p value
	n	Median (IR)	Mean ± SD (CI 95%)	Median (IR)	Mean ± SD (CI 95%)	Median (IR)	Mean ± SD (CI 95%)	
**Consultations** (per patient/year)	90							
Primary care		1,50 (0,00; 3,00)	1,66±1,43 (1,36; 1,96)	2,00 (0,00; 3,00)	2,02±1,74 (1,66; 2,39)	2,30 (0,00; 3,30)	2,36±1,73 (2,00; 2,72)*	0,012
Specialized care		0,00 (0,00; 1,00)	0,60±0,93 (0,40; 0,80)	0,00 (0,00; 1,00)	0,54±0,86 (0,36; 0,72)	0,00 (0,00; 1,00)	0,48±0,86 (0,30; 0,66)	0,238
Emergency care		1,70 (1,00; 2,47)	1,70±1,38 (1,43; 2,00)	1,00 (0,00; 2,00)	1,17±1,29 (0,90; 1,43)*	1,00 (0,30; 1,30)	1,06±0,81 (0,89; 1,23)*	0,002
								
**AH Drugs**	82	30,00 (18,00; 48,00)	33,95±20,84 (29,37; 38,53)	30,00 (18,00; 42,00)	36,68±27,82 (30,56; 42,79)	30,00 (18,00; 42,00)	35,7±24,42 (30,34; 41,07)	0,507
(mg/day) adjusted								
								

* p value <0.05 compared with the pre-PC period;

IR = interquartile range (1st quartile; 3rd quartile); SD = Standard Deviation; CI = confidence interval to 95%; AH = Antihypertensive.

**Fig 3:**
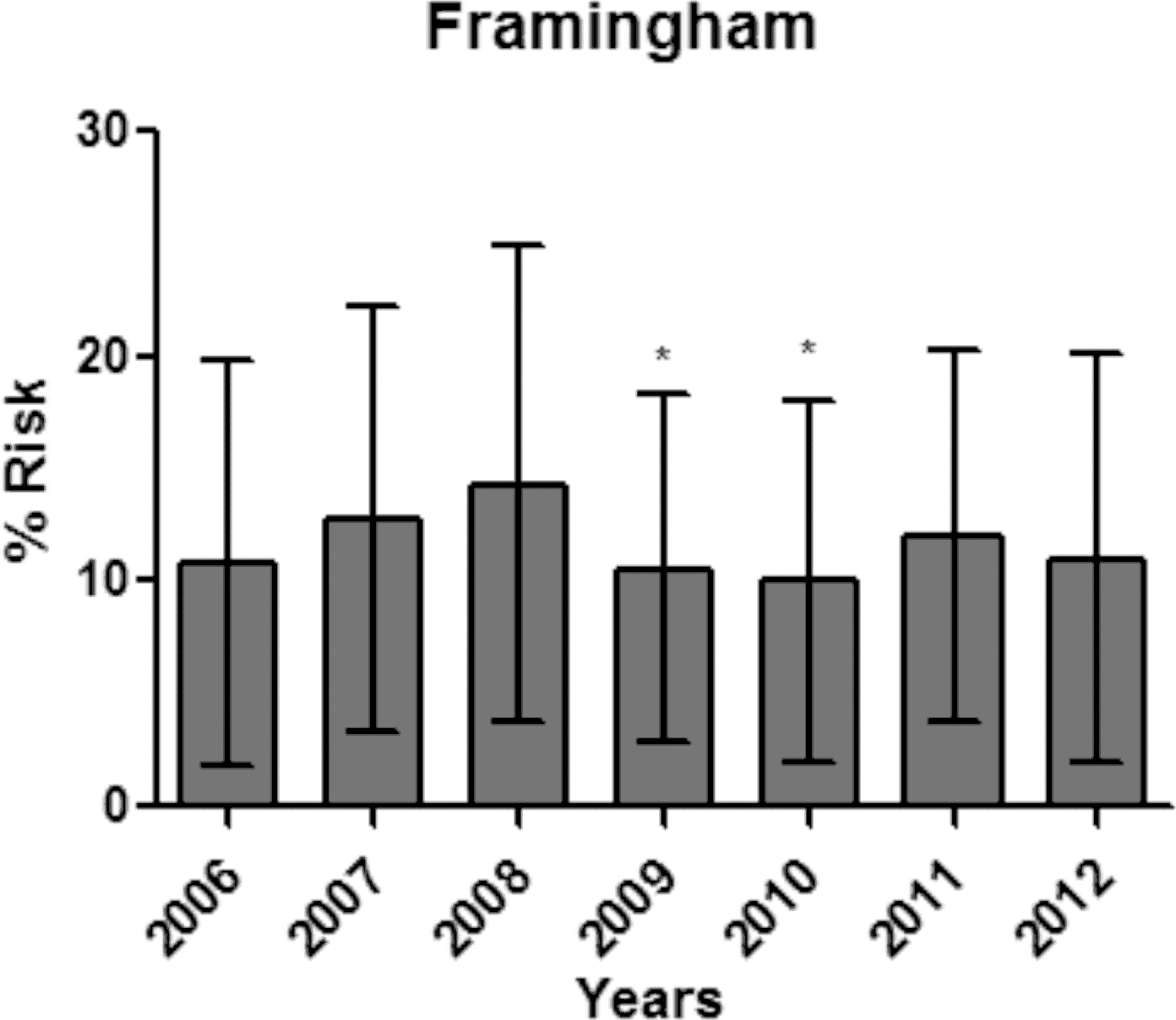
Percentage of average annual coronary risk calculated by the Framingham Risk Scale over the seven year follow-up of hypertensive patients. * p value <0.05 compared with the 2008 year.

The average coronary risk of patients followed was increasing every year in the pre-PC period, 10.8% ± 9.0; 12.8% ± 9.4; 14.3% ± 10.6 in 2006, 2007 and 2008 respectively. From 2009 this trend was interrupted, presenting 10.6% ± 7.7. The average of the three years of the post-PC period was 10.9% ± 7.9 (Fig 3).

## Discussion

As predicted by the literature [33], studies of up to two years are inadequate to assess the effect of changes caused by the management of SAH. In this sense, our continued study by the prolonged period of three years after discharge of patients was relevant to reveal significant results and is consistent with the pharmaceutical intervention. An estimated discontinuance of 40–56% of patients of pharmacotherapeutic monitoring is a problem attributed to this type of study [11, 17]. In our study it was 45.5% and in accordance with the sample size calculation for the dependent variables, SBP and DBP, the 57 patients were representative to make inferences for the study population.

In addition, for the sample study, patients with a prevalent socio-demographic profile of female, elderly, white skin, with only elementary school completed, in occupational activity and lower middle class were estimated. Additionally, there is a cardiovascular risk profile that justifies the prevention of coronary risk, and more than half were classified as obese and 80.8% had a waist circumference above the recommended value. In accordance with the literature it is possible to affirm that the profile of the patients in this study has characteristics representative of the hypertensive population worldwide [3, 4, 30]. Therefore it is possible to extrapolate the results achieved by PC.

Considering blood pressure, PC was able to reduce the mean SBP and DBP, and in the analysis of standard deviation it is observed that there was less variation between SBP and DBP among patients in this period. PC achieved the reduction in mean SBP and DBP of 17.3 and 8.5 mmHg in the PC period. In a study evaluating the outcomes of PC for hypertensive patients in Brazil [11], results were found similar to those achieved by the PC in our study, including the reduction of 18.0 and 12.0 mmHg for SBP and DBP, respectively [11, 34]. Additionally, our study showed that the reduction of blood pressure was maintained at 11.5 and 5.7 mmHg in the post-PC period when compared with the average of the pre-PC period. It is noteworthy that the reduction in blood pressure was statistically significant, because the reduction was greater than 10 mmHg, predicted as significant by the sample calculation.

It was shown in a double-blind randomized study that enalapril reduced SBP and DBP by 8.0 and 3.0 mmHg, respectively [35]. Comparing these results with those obtained in our study, PC was able to reduce SBP and DBP by 9.3 and 5.5 mmHg, respectively, exceeding the reduction obtained by enalapril alone. Our study also showed that in the PC period there was a reduction in the proportion of patients with unsatisfactory SBP and DBP levels, highlighting a trend of clinical control related to these indicators in the post-PC period. There was evidence that PC was associated with the achievement of satisfactory blood pressure for 98.2% of the patients, which remained at 93.0% after PC discharge. It is noteworthy that this rate is higher than that obtained by some antihypertensive drugs, for example, enalapril, which reached 88% [36]. This is relevant for the health technologies for blood pressure control because PC represents greater therapeutic safety compared to pharmacological therapies [37].

PC addresses the individual as a whole in their biopsychosocial characteristics, involving health education activities. Despite using pharmacotherapy in the planning of PC actions, PC is not restricted to the pharmacological effects. In this sense, non-pharmacological interventions and guidelines empowering the patient to their care are made, improving adherence and quality of life [14]. Our results can exemplify this, as PC had the ability to promote from 54% to 100% adherence to pharmacotherapy by the patients. It is noteworthy that the pharmacist improves adherence to pharmacotherapy [38]. In addition, there was an increase in the score in the eight domains of the SF-36 (functional capacity, physical aspects, pain, general health, vitality, social aspects, emotional aspects, and mental health), meaning on average an increase from 66.8 to 85.3 points and a 27.6% improvement in the quality of life of these patients.

As for lipid levels, it was clear that TC was an indicator that had PC as a factor associated with its control and the progressive improvement of its profile in the post-PC period. In our study, PC decreased average TC by 10.6 mg/dL (5%), and evidenced a reduction of 15.3 mg/dL (8%) in post-PC in relation to pre-PC. An experimental study [39] showed that PC for dyslipidemic patients is able to promote the mean difference in TC of 25.6 mg/dL (15% reduction) compared to the control group. Compared to drug treatment for hyperlipidemia, fluvastatin and simvastatin can reduce an average of 16% and 25% TC respectively [31, 40]. It is noteworthy that three years after PC our study showed a TC reduction of 3% more on average, showing thereby that the effect of PC measured by this indicator was progressive. That was crucial to the increase in the proportion of patients with satisfactory values for TC from 27.3% to 51.5% in the PC period and also to 60.6% in the post-PC period.

The improved clinical indicators directly help to reduce the risk associated with coronary heart disease. As justified by the researchers [41], the effectiveness of PC in improving outcomes resulted in the reduction from 10.6% to 7.7% in the coronary risk [42, 43]. It is important to emphasize that in our study, the mean coronary risk had a reduction in the PC period and the first year post-PC compared to the last pre-PC year, 2008. PC reduced coronary risk by 26%, i.e. 3.77% in the Framingham score which represented a reduction of risk in 92% of patients classified as high coronary risk and 60% of medium risk patients, leading to 34% of these patients to low risk. It is possible to point out that 4.8% of patients were smokers and 18.3% were diabetics and that these factors did not change during the study. Also, because age is a coronary risk factor for hypertensive patients, the trend was that under the same clinical parameters these patients had increased coronary risk over the seven years, from 2006 to 2012. This fact makes it possible to note that the modification of coronary risk is strongly associated with improved lipid values and the control of arterial blood pressure by PC [26, 27, 30].

Regarding the care indicators, there was evidence of the increase in the annual average number of consultations per patient in primary care during the post-PC period. We highlight that in this period the average of the consultations was closer to the median, and the median was closer to the third quartile, this represents that more than 50% of patients had the number of consultations in primary care increased after PC. In addition, in the pre-PC period 75% of the patients had two or more consultations in emergency care, and in later periods this percentage decreased to 50%, half of patients had one or less consultation in emergency care. It should be noted that there was a higher proportion of patients with the number of consultations equal to or less than one in emergency care. This may be associated to the possible reduction of hypertensive crises due to blood pressure control achieved by PC.

Our study showed that PC has promoted primary medical care for patients. In this context it important to emphasize that for chronic non-communicable diseases such as SAH, re-education is fundamental for daily and preventive care, and this often occurs in primary care [4]. Although the literature reveals that PC improves outcomes and changes the consumption of drugs, justifying this by health education, optimization of pharmacotherapy and improved treatment adherence, there was no evidence in the change in drug consumption in our study sample [4, 44, 45].

Among the limitations of this study we highlight that many patients had missing data. This could have reduced the power of the statistical tests we used for LDL, HDL and TG analysis. However, even with these missing data, we found evidence of improvement in SBP and DBP indicators, TC and healthcare indicators. In addition, due to the study design, the absence of a control group can interfere in the results, because some modifications in the municipality health assistance such as new medicine standard in the PHS or some specific hypertension government care program introduced after 2008 could influence the improvement of the indicators analyzed. Thus, we highlight there were not changes in this sense despite spironolactone and losartan being introduced into the PHS from 2010 and 2011, respectively, because the Cochran Q test is sensitive to the indicators for trend analysis over time, and we performed it for three years before and three years after intervention.

## Conclusion

Our results showed that PC was effective in blood pressure control and control of TC, representing an effectiveness that was not time specific, that is, PC also contributed to the long-term control of these clinical indicators. Thus, we show that the improved outcomes achieved by PC, mainly related to blood pressure, were able to decrease cardiovascular risk and promote further preventive care for the patient. Our study showed that in one year the PC program has been able to achieve the adherence of patients and improve life quality. To summarize, this study shows that PC is a professional practice able to be incorporated into the health services in order to contribute to the reduction of the risk of coronary heart disease and also morbidity and mortality from hypertension, and the results are maintained even three years after discharge.

## Supporting Information

S1 Appendix TREND Statement Checklist—adapting the concepts of quiasi-experimental studies by the authors. (PDF)
