# Supplementary material for: Impact Assessment of Pharmaceutical Care in the Management of Hypertension and Coronary Risk Factors after Discharge
Source: PLoS One. 2016 Jun 15;11(6):e0155204. doi: 10.1371/journal.pone.0155204 (PMC4909302; doi:10.1371/journal.pone.0155204)
Supplement: S1 Appendix — (PDF) [file pone.0155204.s001.pdf]

S1 Appendix
TREND Statement Checklist - adapting the concepts of quiasi-experimental studies by the authors.

TREND Statement Checklist - Impact assessment of pharmaceutical care in the management of hypertension and coronary risk factors after discharge

Paper Section/ Topic	Item No	Descriptor	Reported?	Pag
**Pg #**				
**Title and Abstract**				1 - Impact assessment of pharmaceutical care in the management of hypertension and coronary risk over three yaers after discharge
Title and Abstract	1	Information on how unit were allocated to interventions		1- The PC was performed in two basic units of the public health system in Ribeirão Preto-SP, Brazil, where the pharmacist followed up 104 patients
		Structured abstract recommended		
		Information on target population or study sample		1- 104 hypertensive patients. The clinical indicators of systolic (SBP) and diastolic blood pressure (DBP), triglycerides, total-cholesterol, high and low density lipoprotein cholesterol were raised, as well as care indicators related to the number of consultations (basic, specialized and emergency care) and antihypertensive drugs used.
**Introduction**				
Background	2	Scientific background and explanation of rationale		2-3 - We emphasize the importance of chronic diseases in the world, the high prevalence of hypertension, ineffective results for the control of blood pressure and called it to the results that the PC has achieved, and highlight the importance of showing these results after discharge, as our study

aimed.

Theories used in designing behavioral interventions

Methods

Participants 3

Eligibility criteria for participants, including criteria at different levels in recruitment/sampling plan (e.g., cities, clinics, subjects)

3 - Individuals diagnosed with SAH and in medical care for the disease, aged over 20 years, users of the health unit within the PC program, and those who used at least one antihypertensive medication were included in the program. The program excluded patients who could not continue the planning of pharmaceutical consultations, pregnant women and those who had some kind of diagnosed cognitive impairment.

4- From August to November 2008 the patient was approached at the time of receipt of the drug in the health unit, and invited to participate in the PC program. If the patient met the inclusion criteria and did not meet any item in the exclusion criteria, they were invited to sign the Free and Informed Consent Terms, with guidance from the pharmacist of the program.

4- From this moment the patient was included in the PC program. During 2009, from January to December, one pharmacist followed up 104 patients in this PC program. In 2013, data collection for this study was performed in order to gather the clinical and health care data of individuals monitored by PC. Thus, from January 2006 to December 2012 the data were collected through the patient record and the Hygiaweb® computerized system.

Method of recruitment (e.g., referral, self-selection), including the sampling method if a systematic sampling plan

was implemented			
Recruitment setting			3- in two units of primary health of the PHS in Ribeirão Preto-SP, Brazil ; 4- approached at the time of receipt of the drug in the health unit, and invited to participate
Settings and locations where the data were collected			
Interventions	4	Details of the interventions intended for each study condition and how and when they were actually administered, specifically including:	5 - Therefore, during 2009 each patient was scheduled to consult the pharmacist once a month during a year in the health units of study.
o Content: what was given?			4 - Subsequent consultations followed the relevant activities of pharmacotherapeutic monitoring, considering blood pressure measurements and measures of cardiovascular risk, analysis of medicines and test results, health education with guidance on patient behavior regarding life habits, adherence to treatment, and if necessary interventions in pharmacotherapy.
o Delivery method: how was the content given?			
o Unit of delivery: how were the subjects grouped during delivery?			5- Therefore, during 2009 each patient was scheduled to consult the pharmacist once a month during a year
o Deliverer: who delivered the intervention?			3- This PC program was conducted by one pharmacist in two units of primary health of the PHS in Ribeirão Preto-SP, Brazil ; 5- the pharmacist

○ Setting: where was the intervention delivered?			5- in the health units of study
○ Exposure quantity and duration: how many sessions or episodes or events were intended to be delivered? How long were they intended to last?			4- was scheduled to consult the pharmacist once a month during a year
○ Time span: how long was it intended to take to deliver the intervention to each unit?			4- one year
○ Activities to increase compliance or adherence (e.g., incentives)			5- During all PC period, the pharmacist worked on health education through informative lectures, educational materials on health, and guidance during the consultations. Adherence to drug therapy was also worked on.
Objectives	5	Specific objectives and hypotheses	7- Indicators to assess the impact of Pharmaceutical Care; under hypothesis that the results are maintained even three years after discharge
Outcomes	6	Clearly defined primary and secondary outcome measures	7- The indicators were defined according to the clinical and care data, considering primary outcome, blood pressure, and secondary outcomes, plasma lipid levels, coronary risk and care.
Methods used to collect data and any methods used to enhance the quality of measurements			4- Thus, from January 2006 to December 2012 the data were collected through the patient record and the *Hygiaweb*® computerized system.
Information on validated instruments such as psychometric and biometric properties			
Sample Size	7	How sample size was determined	6- Sample planning and sample of Pharmaceutical Care

		and, when applicable, explanation of any interim analyses and stopping rules	study
Assignment Method	8	Unit of assignment (the unit being assigned to study condition, e.g., individual, group, community)	8 – group - Importantly, the data were divided into three periods for analysis: from 2006 to 2008, defined as pre-PC; in 2009 defined as PC; from 2010 to 2012 defined as post-PC.
Blinding (masking)	9	Whether or not participants, those administering the interventions, and those assessing the outcomes were blinded to study condition assignment; if so, statement regarding how the blinding was accomplished and how it was assessed.	No apllied
Unit of Analysis	10	Description of the smallest unit that is being analyzed to assess	7- For the analysis of clinical indicators, the data were categorized as satisfactory;

		intervention effects (e.g., individual, group, or community)	and unsatisfactory. Pg 6 and 8 – Coronary risk – Framingham scale; Care indicators – drugs in mg/ patient / years; and consulation: countable consultations.
If the unit of analysis differs from the unit of assignment, the analytical method used to account for this (e.g., adjusting the standard error estimates by the design effect or using multilevel analysis)			
Statistical Methods	11	Statistical methods used to compare study groups for primary methods outcome(s), including complex methods of correlated data	8-9 - The inferential statistical analysis was performed using *Statistical Analysis System* (SAS) version 9.2 and to develop the graphs statistical analysis *GraphPad Prisma* version 5 was used. For hypothesis testing a 5% significance level was considered. Importantly, the data were divided into three periods for analysis: from 2006 to 2008, defined as pre-PC; in 2009 defined as PC; from 2010 to 2012 defined as post-PC. The inferential statistic was based on paired data, this means relating to the data of the same individuals for analysis at different time points, because of this there were no potential confounders. Thus, for the clinical indicators the *Cochran Q test* was performed to compare variables of categorical type. ANOVA for medication and coronary risk and Friedman for consultation.
Statistical methods used for additional analyses, such as a subgroup analyses and adjusted analysis			
Methods for imputing missing data, if used			
Statistical software or programs used			
Method used to assign units to study conditions, including details of any restriction (e.g., blocking, stratification, minimization)			Pg 7 - Figure 1.

Inclusion of aspects employed to help minimize potential bias induced due to non-randomization (e.g., matching)			Pg 7 -8 – The choice of the analysis methods.
**Results**			
Participant flow	12	Flow of participants through each stage of the study: enrollment, assignment, allocation, and intervention exposure, follow-up, analysis (a diagram is strongly recommended)	We believe we represent the flow of patients in Figure 1. We put on methods due justify the stratification of the sample size for each indicator analyzed and described this methodology. This option to put the methodology is justified by this study does not refer specifically to the intervention program, but the period after discharge. However, we discussed this in the discussion of the results.
○ Enrollment: the numbers of participants screened for eligibility, found to be eligible or not eligible, declined to be enrolled, and enrolled in the study			Pg 6 - The method used to select the sample was convenience sampling, whereby 191 patients were invited who were considered eligible in accordance with the inclusion criteria and 37 individuals were exclude because did not fulfill the eligibility criteria for this study;
○ Assignment: the numbers of participants assigned to a study condition			Pg 6 - Figure 1 – 104 patients, after before comparasion.
○ Allocation and intervention exposure: the number of participants assigned to each study condition and the number of participants who received each intervention			Pg 6 - whereby 191 patients were invited who were considered eligible, and 104 completed the follow-up. Figure 1.

○ Follow-up: the number of participants who completed the follow-up or did not complete the follow-up (i.e., lost to follow-up), by study condition			pg 11- According to the sample size calculation for the dependent variable, the 57 patients who had the SBP and DBP variable analyzed in our study were representative to make inferences to the study population.
○ Analysis: the number of participants included in or excluded from the main analysis, by study condition			
Description of protocol deviations from study as planned, along with reasons			Pg 4- From this moment the patient was included in the PC program. During 2009, from January to December, one pharmacist followed up 104 patients in this PC program. In 2013, data collection for this study was performed in order to gather the clinical and health care data of individuals monitored by PC.
Recruitment	13	Dates defining the periods of recruitment and follow-up	
Baseline Data	14	Baseline demographic and clinical characteristics of participants in each study condition	Pg 9 table 1.
Baseline characteristics for each study condition relevant to specific disease prevention research			Pg 10 - Table 2.

Baseline comparisons of those lost to follow-up and those retained, overall and by study condition			Not applied.
Comparison between study population at baseline and target population of interest			Pg 10 – 11 -Table 2; table 3 and figure 2 and 3.
Baseline equivalence	15	Data on study group equivalence at baseline and statistical methods used to control for baseline differences	Pg 10 – 11 -Table 2; table 3 and figure 2 and 3.
Numbers analyzed	16	Number of participants (denominator) included in each analysis for each study condition, particularly when the denominators change for different outcomes; statement of the results in absolute numbers when feasible	Pg 10 – 11 – table 2 and 3.
Indication of whether the analysis strategy was “intention to treat” or, if not, description of how non-compliers were treated in the analyses			
Outcomes and estimation	17	For each primary and secondary outcome, a summary of results for each estimation study condition, and the estimated effect size and a confidence interval to indicate the precision	Pg 10 – 11 -Table 2; table 3 and figure 2 and 3.

Inclusion of null and negative findings			Pg 10 - 11 -Table 2; LDL, HDL, TG; and table 3 – medication.
Inclusion of results from testing pre-specified causal pathways through which the intervention was intended to operate, if any			
Ancillary analyses	18	Summary of other analyses performed, including subgroup or restricted analyses, indicating which are pre-specified or exploratory	*Pg 11 – Table 3; Post test bonferroni and Dunns.* Not applied.
Adverse events	19	Summary of all important adverse events or unintended effects in each study condition (including summary measures, effect size estimates, and confidence intervals)	Not applied.
**DISCUSSION**			
Interpretation	20	Interpretation of the results, taking into account study hypotheses, sources of potential bias, imprecision of measures, multiplicative analyses, and other limitations or weaknesses of the study	Pg 11 – 14- We discussed the results of each indicator and compare with the literature, the limitations were explained in the last paragraph of the manuscript, and concluded by stating that there is a hypothesis generated, because this study is quasi-experimental.

Discussion of results taking into account the mechanism by which the intervention was intended to work (causal pathways) or alternative mechanisms or explanations	Pg 13 - PC addresses the individual as a whole in their biopsychosocial characteristics, involving health- education activities. Despite using pharmacotherapy in the planning of PC actions, PC is not restricted to the pharmacological effects. In this sense, non-pharmacological interventions and guidelines empowering the patient to their care are made, improving adherence and quality of life.
Discussion of the success of and barriers to implementing the intervention, fidelity of implementation	Pg 16 - . To sum up, these results showed that PC is a professional practice able to be incorporated into the health services in order to contribute to the reduction of the risk of coronary heart disease and also to morbi-mortality from hypertension, under the hypothesis generated by this work that the results are maintained even three years after discharge.
Discussion of research, programmatic, or policy implications	Pg 15 - is expected that these results help in the development of systematic reviews and methodological development of new studies evaluating this technology in health. Considering the optimization of outcomes by PC, which are also supported by other studies, it is intended that these results assist in the effective implementation of PC in the PHS, mainly in the collaboration of not only the immediate outcomes, but also for viewing the PC results after discharge of patients.

Generalizability	21	Generalizability (external validity) of the trial findings, taking into account the study population, the characteristics of the intervention, length of follow-up, incentives, compliance rates, specific sites/settings involved in the study, and other contextual issues	Pg 11- According to the sample size calculation for the dependent variable, the 57 patients who had the SBP and DBP variable analyzed in our study were representative to make inferences to the study population.;<br><br>Pg 12 - Thus, the sample treated in this study has the characteristic profile of a patient that is representative of the hypertensive population worldwide. Therefore it is possible to extrapolate the results achieved by PC as for clinical and care indicators, and the risk percentage to coronary heart disease.
Overall Evidence	22	General interpretation of the results in the context of current evidence and current theory	Pg 15 - However, it is possible to emphasize that for the SBP and DBP indicators the sample size was consistent with the calculated, as well as the TC and healthcare indicators.

From: Des Jarlais, D. C., Lyles, C., Crepaz, N., & the Trend Group (2004). Improving the reporting quality of nonrandomized evaluations of behavioral and public health interventions: The TREND statement. *American Journal of Public Health*, 94, 361-366.
